# Developmental timing of flash drought influences offspring survival in the field

**DOI:** 10.1007/s00442-025-05702-7

**Published:** 2025-04-17

**Authors:** Ayley L. Shortridge, Morgan A. Clark, Paulette Gutierrez, Caleb J. Krueger, Fredric J. Janzen

**Affiliations:** 1 Ecology, Evolution, & Behavior Program, Department of Integrative Biology, W. K. Kellogg Biological Station, Michigan State University, Hickory Corners, MI USA; 2 Ecology, Evolution, & Behavior Program, Department of Fisheries & Wildlife, W. K. Kellogg Biological Station, Michigan State University, Hickory Corners, MI USA

**Keywords:** Climate change, Physiology, Embryonic development, Hydric stress, Vertebrate

## Abstract

Extreme climate events, including rapid-onset flash droughts, are increasing with anthropogenic climate change. Flash droughts impact growth and survival of plants, microbes, and invertebrates, yet less is known about their ecological consequences in vertebrates. Although constant water deprivation during vertebrate embryonic development influences a range of offspring traits, the effects of acute, short-term hydric stress are less well-studied, particularly in the context of subsequent survival in the field. In this study, we combined experimental manipulation in the laboratory with a large-scale field experiment to examine the effects of the developmental timing of flash drought in an oviparous vertebrate model. We exposed common snapping turtle (*Chelydra serpentina*) eggs to simulated flash drought events at different stages of embryonic development and measured the effects on growth and offspring phenotypes. We then conducted an experimental release in the field to evaluate offspring survival during migration from the nest. Flash drought during mid-to-late development decreased egg mass, incubation time, and hatchling body size, while flash drought during late development substantially limited post-hatching survival in the field. This study is among the first to examine juvenile survival effects of flash drought during embryonic development in a vertebrate system. Our results suggest that early-life mortality is likely to increase as flash droughts intensify with climate change. This study contributes to a growing body of research on the ecological consequences of extreme climate events and highlights the importance of considering these events in a developmental context.

**Supplementary Information:**

The online version contains supplementary material available at 10.1007/s00442-025-05702-7.

## Introduction

Soil moisture is a key limiting factor in the survival, growth, and function of a wide range of organisms. The biotic impacts of soil water availability have been widely documented in many taxa, including plants, microbes, and invertebrates (Yan et al. 2018; Pugnaire et al. 2019; Xu et al. 2019; Flórián et al. 2019; Evans et al. 2022), yet less is known about the effects of soil moisture on vertebrates. Ground-nesting oviparous vertebrates frequently experience variable hydric conditions in the nest (e.g., Packard et al. 1985; Rivas et al. 2018). For instance, nest flooding and desiccation often lead to egg mortality in birds and non-avian reptiles (e.g., Cassill and Watkins 2022; Elas et al. 2023). Embryos that survive hydric stress may respond through developmental plasticity, leading to phenotypic variation that may impact future survival and fitness (West-Eberhard 2003; Sommer 2020).

Climate change is driving an increase in hydric extremes, including unprecedented drought events worldwide (Satoh et al. 2022). Drought is broadly defined as a period of water deficit, typically caused by a combination of low precipitation and high evapotranspiration (Ault 2020). While droughts can develop over months or years, rapid-onset “flash droughts,” which occur on a subseasonal time scale, are increasingly prevalent (Yuan et al. 2023). Flash droughts can rapidly deplete soil moisture, suggesting that these events have the potential to impact the course of embryonic development in ground-nesting vertebrates.

Turtles (order Testudines) are oviparous and typically nest in soil, making them an excellent vertebrate model system for studying the effects of soil moisture during egg incubation. Many species lay flexible-shelled eggs, which are highly sensitive to changing soil moisture due to a porous mineral layer (Packard et al. 1982; Packard 1991; Ackerman and Lott 2004). Even so, the potential impacts of flash drought on offspring phenotype and fitness in turtles remain unknown. Turtles are among the most threatened vertebrate taxa globally, with over half of all species listed as threatened and over one-third listed as endangered under IUCN criteria (Stanford et al. 2020). As flash drought and other extreme climate events intensify under climate change, research is needed to understand how drought-induced changes in soil moisture affect turtle embryonic development and survival.

Previous field and laboratory studies have identified phenotypic effects of substrate moisture during egg incubation in reptiles, although not necessarily in the context of climate change (Morris et al. 1983; Janzen et al. 1995; Packard 1991, 1999). Flexible-shelled eggs generally absorb water in wetter conditions and lose water in drier conditions, impacting the reserve of water available to the developing embryo (Packard 1999). Dry incubation conditions limit embryonic metabolism and growth, thereby reducing egg mass, hatching success, incubation time, and hatchling size (Packard et al. 1981; Morris et al. 1983; Tucker and Paukstis 2000). Additionally, turtle hatchlings incubated in constant dry conditions have lower body water content and desiccation tolerance (Finkler 1999), reduced movement speed (Miller et al. 1987; Miller 1993), and may travel shorter overland distances each day (Finkler et al. 2000).

Soil moisture during turtle embryonic development has been suggested to affect post-hatching fitness, but few studies have tested this hypothesis. Turtle hatchling survival often varies positively with body size (Janzen 1993; Janzen et al. 2000a, b; Tucker 2000; but see Congdon et al. 1999; Kolbe and Janzen 2001; Filoramo and Janzen 2002). If “bigger is usually better” (Rollinson and Rowe 2015), then the smaller body size imparted by constant dry incubation conditions should incur a fitness cost. Hatchlings incubated in dry laboratory conditions are slower and more vulnerable to dehydration, traits which may also be associated with reduced fitness (Miller et al. 1987; Finkler 1999).

The timing of hydric stress may be an important factor in turtle embryonic development. The hydric environment in natural nests is not constant; soil moisture varies over time in response to rainfall and evaporation (e.g., Packard et al. 1985). In two previous studies, hatchling phenotypes were driven primarily by the hydric environment during the last two-thirds of incubation, possibly due to “compensatory water exchange,” or increased water absorption when eggs transitioned from dry to wet conditions during mid-development (Gutzke and Packard 1986; Delmas et al. 2008; but see Packard and Packard 1988). As flash droughts become more frequent and intense under climate change, it becomes critical to investigate how short-term substantive reductions in soil moisture impact neonatal turtle development, hatchling phenotype, and early-life fitness.

In this study, we use a combination of laboratory and field methods to determine how flash drought during embryonic development affects early-life phenotypes and survival in the common snapping turtle, *Chelydra serpentina*. This species lays large clutches of flexible-shelled eggs. For this study, we focused on a *C. serpentina* population that nests in sand prairie habitat along the Mississippi River. The central United States, including our study site, is considered a “global hotspot” for flash drought (Christian et al. 2021). Most flash drought events in this region occur between June and August (Christian et al. 2019), coinciding with the nesting and incubation phenology of *C. serpentina* (St Juliana and Janzen 2007). In this system, offspring experience strong selection during early life stages, including high predation during the hatchling dispersal stage as they emerge from the nest and move toward water. Thus, potential fitness differences imposed by the nest environment should be observable during this life stage (Janzen 1993).

We predicted that experimental flash drought would negatively impact *C. serpentina* embryo growth and hatchling survival. Specifically, we expected that flash drought imposed at any time during embryonic development would negatively affect water flux between eggs and substrate, causing eggs to lose mass. We also predicted that any mass lost during early incubation would be regained by the time of hatching due to compensatory water exchange, while mass lost during mid-to-late incubation would not be regained. Finally, we expected that flash drought during mid-to-late incubation would reduce hatching success, incubation time, body size, and hatchling survival, while increasing time to disperse and mass lost during dispersal from the nest to the water.

## Materials and methods

### Egg collection and incubation

Between 02-Jun and 06-Jun 2022, we collected 20 eggs from each of 10 *C. serpentina* nests at Thomson Sand Prairie in Carroll County, IL within a few hours of deposition (N = 200 eggs). We used a permanent marker to label each egg with a unique code identifying the individual and clutch of origin. We then placed eggs in Styrofoam containers and covered them with a layer of moist sand. On 06-Jun-2022, we transported all eggs to our laboratory. We weighed eggs to the nearest 0.01 g and randomly assigned them to one of five treatment groups, beginning experimental incubation on 07-Jun-2022. The treatment groups were exposed to a simulated flash drought event during early, middle, late, or mid + late incubation, while the control group experienced no drought (Table 1). Each clutch was equally represented in all treatments. We placed eggs in covered plastic boxes filled with vermiculite substrate, arrayed in 4 × 5 matrices, then placed the boxes in a Percival incubator set to a daily cycle of 12 h 28 °C/12 h 31 °C, representing a typical diel range in the center of nests at this site (Kolbe and Janzen 2001; Telemeco et al. 2016). This temperature regime produces 100% female offspring for this population of *C. serpentina* (Janzen 2008; Krueger and Janzen 2022), thereby eliminating gonadal sex as a confounding treatment factor.

**Table 1:** Summary of the five treatment groups used in this experiment

Treatment	T1: Day 1–21	T2: Day 22–42	T3: Day 43–63
Control	–	–	–
Early	Drought	–	–
Middle	–	Drought	–
Late	–	–	Drought
Mid + late	–	Drought	Drought

Under these experimental conditions, we estimated incubation time to be approximately nine weeks, which we subdivided into three 21-day segments. We maintained the control group in moist vermiculite (water potential = −150 kPa) throughout the incubation period, representing a typical non-drought hydric environment (Packard et al. 1985; but see Ackerman and Lott 2004). To simulate drought conditions, we placed the other four groups in drier vermiculite (water potential = −850 kPa) for 3–6 weeks during early, middle, late, or mid + late incubation (Table 1).

Each week, we individually weighed eggs to the nearest 0.01 g, rehydrated the vermiculite in boxes to maintain target moisture levels, and rotated boxes within the incubator to minimize thermal gradient effects. After the first hatchlings were observed on 02-Aug-2022, we monitored boxes daily and recorded pipping and hatching dates for each egg. We weighed hatchlings to the nearest 0.01 g approximately 24 h after hatching, and measured carapace and plastron length and width to the nearest 0.01 mm. Hatchlings were housed individually in small plastic tubs with a piece of damp paper towel.

Once all hatchlings emerged on 13-Aug-2022, we divided them into four release groups, such that treatment was equally represented in each group. As *C. serpentina* hatchlings can be identified by their unique plastron markings (Janzen et al. 2000b), we photographed the plastron of each hatchling for later identification. Each release group was further identified with a small notch on a marginal scute. We then group-housed hatchlings in four plastic boxes lined with damp paper towels, with each treatment represented equally in each group, and transported them to the original collection site on 15-Aug-2022.

### Release experiment

We conducted the release experiment at Thomson Sand Prairie, where eggs were initially collected. We constructed an 80 m drift fence parallel to the Mississippi River using 0.3 m tall aluminum flashing, with 17 pitfall traps placed along the fence at 5 m intervals. To install the pitfall traps, we buried 4.5 L plastic jars with their openings at ground level. We created four release points by digging shallow artificial nest cavities centered at the midpoint of the fence. The release points were 10 m apart and 30 m upslope from the fence, in a sandy patch typical of *C. serpentina* nests at this site (Kolbe and Janzen 2002; pers. obs.). We released all hatchlings at midday on 16-Aug-2022, a typical date and time of day for hatchling emergence in this population (Congdon et al. 1999; Delaney and Janzen 2019). Subsequently, we monitored the drift fence three times a day at 7:00 AM, 1:00 PM, and 7:00 PM and collected all hatchlings captured in the pitfall traps. We identified, weighed to the nearest 0.01 g, and released each recaptured hatchling at the edge of the Mississippi River. After two consecutive days with no hatchlings recovered, we concluded the experiment on 22-Aug-2022.

### Statistical analyses

All statistical analyses were conducted using R version 4.4.1 (R Core Team 2024). Linear and generalized linear mixed models were run using the lme4 package (Bates et al. 2015) unless otherwise noted. To assess changes in egg mass over time, we conducted an analysis of covariance with repeated measures. For this model, treatment and week were fixed effects, initial egg mass was the covariate, and clutch was a random effect. We modeled hatching success as a function of treatment using a logistic regression model, with clutch as a random effect and initial egg mass as a covariate. To model incubation time, we used a linear mixed effects model fit by restricted maximum likelihood (REML), denoting treatment as a fixed effect and clutch as a random effect. Similarly, we modeled hatchling mass, and body size metrics (carapace length, carapace width, plastron length, plastron width) using linear mixed models by REML. In each case, treatment was the predictor variable, initial egg mass was a covariate, and clutch was a random effect.

To analyze hatchling survival during migration from the artificial nests, we first used logistic regression to model recapture as a function of treatment, with hatchling mass as a covariate and clutch as a random effect. We then analyzed survival separately as a function of early, middle, and late drought treatments, again using logistic regression with the covariate and random effect described above. We modeled dispersal time as a function of treatment, with hatchling mass as a covariate, using a linear regression. Dispersal time was not normally distributed, so we used the R package DHARMa (Hartig 2022) to check that our model met the assumptions of a linear regression. Finally, we modeled mass lost during dispersal as a function of treatment using a linear mixed model fit by REML, with hatchling mass as a covariate and clutch as a random effect. Figures were created in ggplot2 (Wickham 2016).

## Results

**Fig. 1:**
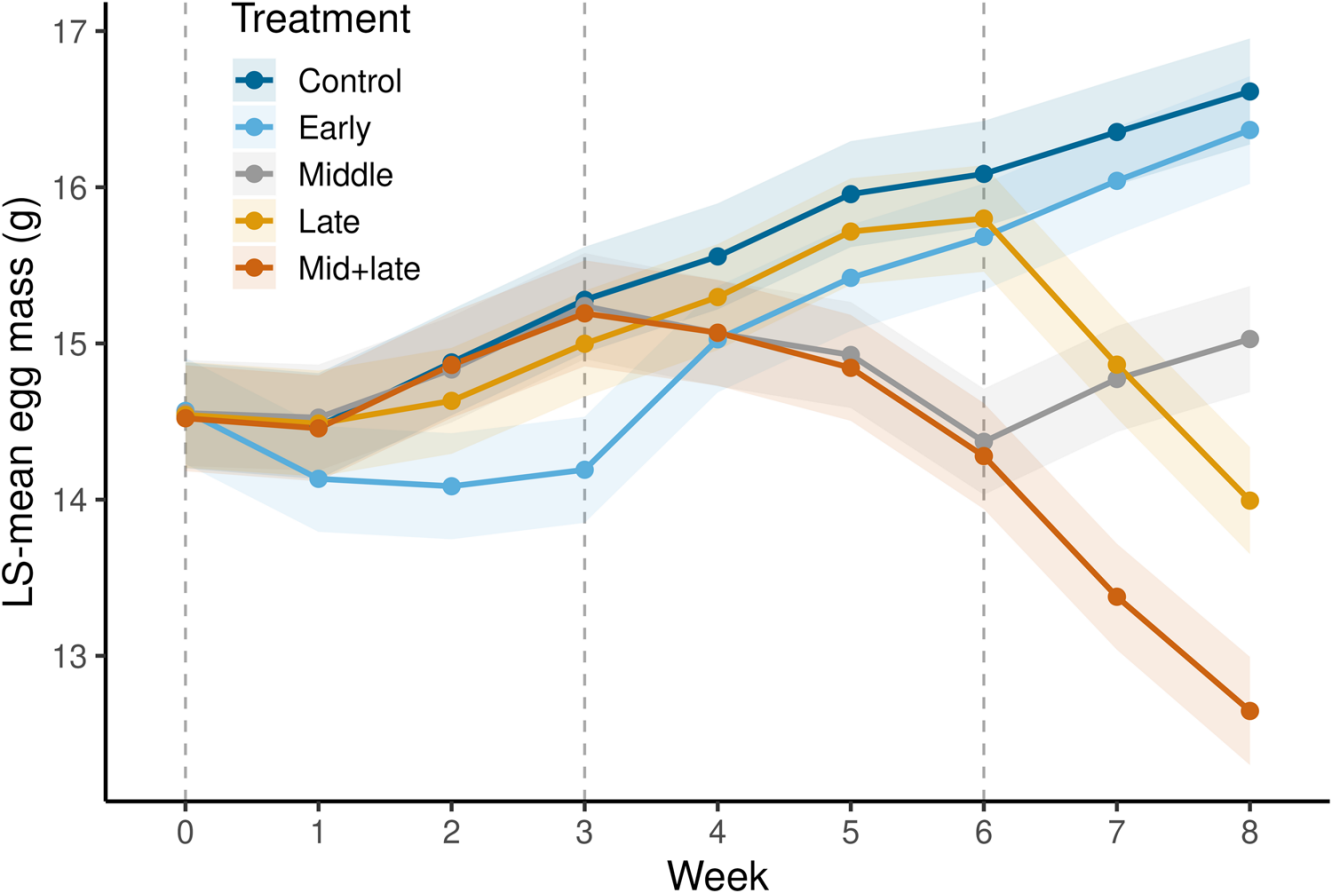
Least squares means and 95% confidence intervals of *Chelydra serpentina* egg mass (g) for each incubation treatment over time (weeks) using an analysis of covariance with repeated measures (N = 200, F = 46.11, P < 0.001). Dashed lines indicate timing of treatment implementation

Egg mass was interactively affected by treatment and time (Fig. 1; Table 2). Overall, eggs in non-drought conditions increased in mass, while eggs in drought conditions decreased in mass. However, eggs in the early drought treatment were able to recover and reach the same average mass as the control group by the end of incubation. By contrast, eggs in the middle, late, and mid + late drought treatments ended incubation at lower mass than the control group, with the mid + late drought treatment being the lowest. At the end of incubation, eggs in the mid + late group weighed 3.93 g (or 23.7%) less on average than the control group eggs.

**Table 2:** Repeated measures analysis of covariance of change in egg mass during incubation

Source of variation	df	Mean square	F-ratio	P-value
Treatment	4	69.8	171.83	< 0.001*
Week	8	21.7	53.50	< 0.001*
Initial egg mass	1	2806.4	6906.36	< 0.001*
Treatment × week	32	18.7	46.11	< 0.001*

Hatching success was uniformly high across treatment groups, with an overall 93.5% hatch rate (N = 187). We found no impact of treatment (F = 1.35, P = 0.25) or egg mass (F = 0.066, P = 0.80) on hatching success. Of the 13 eggs that failed to hatch, two developed to late-term embryos and 11 did not develop. Dissection confirmed that both embryos were gonadal females, consistent with the expected sex under the thermal regime used in this experiment.

**Fig. 2:**
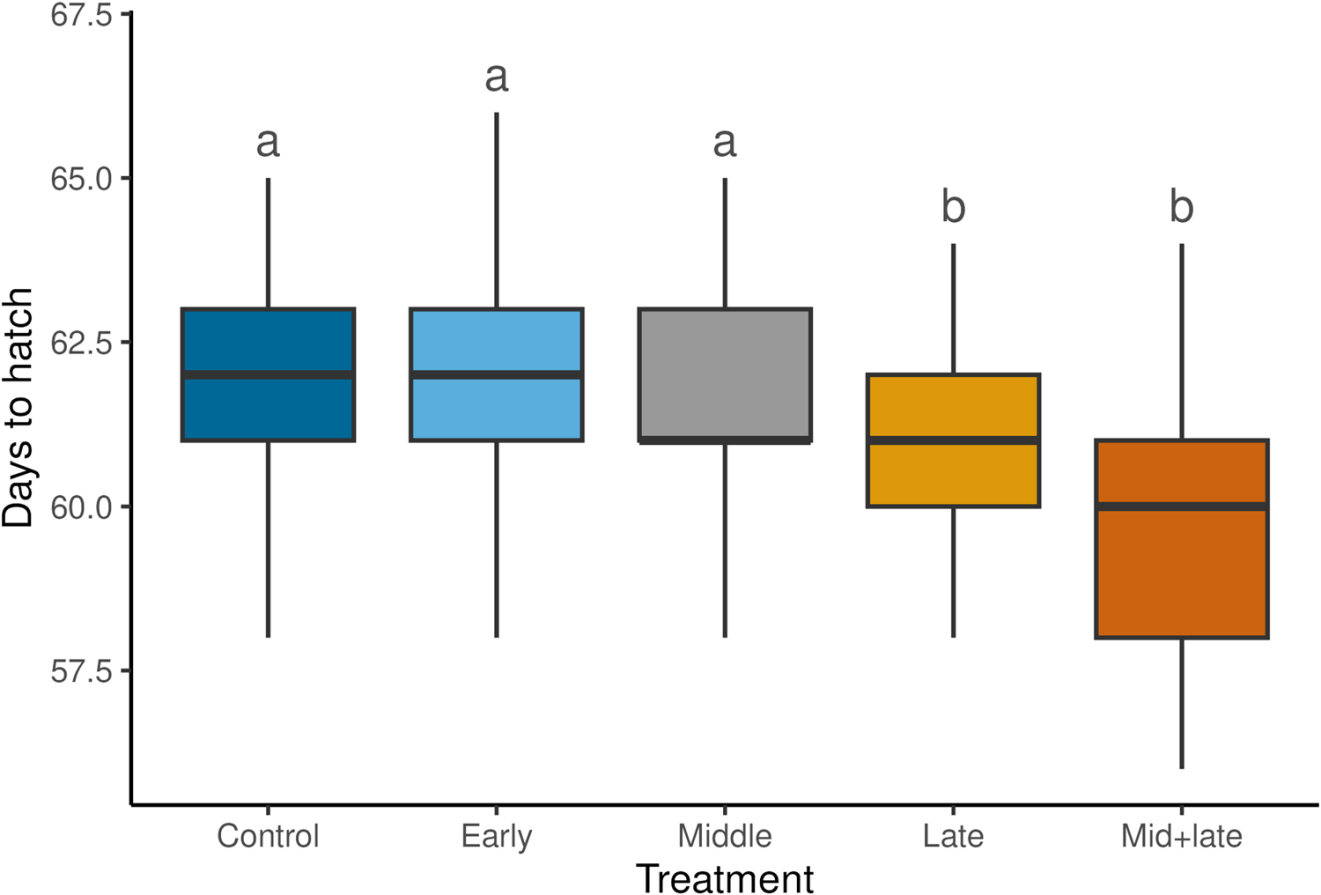
Incubation time (d) by treatment (N = 187). Boxes represent median and interquartile range; vertical lines indicate minimum and maximum values

Incubation time ranged between 56 to 67 days and varied as a function of treatment (Fig. 2; F = 10.9, P < 0.001). Eggs in the late and mid + late drought treatments took less time to hatch on average. Eggs in the late drought group hatched 0.9 d earlier than eggs in the control group, while eggs in the mid + late drought group hatched 2.3 d earlier.

We analyzed five metrics of hatchling body size: hatchling mass (g), carapace length, carapace width, plastron length, and plastron width (mm). Each variable was positively correlated with initial egg mass; eggs with greater mass at the start of incubation produced larger hatchlings, regardless of treatment (Table 3). Drought treatment significantly affected all hatchling size metrics except plastron width (Table 3). Hatchling mass was not affected by early drought; however, drought during middle, late, and mid + late incubation substantially limited hatchling mass. Hatchlings in the mid + late group had the lowest average mass, at 1.50 g (13.0%) lighter than the control group. Similarly, drought during middle, late, and mid + late development reduced carapace length, carapace width, and plastron length.

**Table 3:** F-ratios and levels of significance (in parentheses) for analyses of variance on five metrics of hatchling body size

Variable	Source of variation	
	Treatment	Initial egg mass
Mass	65.06 (< 0.001)*	497.41 (< 0.001)*
Carapace length	10.46 (< 0.001)*	75.30 (< 0.001)*
Carapace width	15.87 (< 0.001)*	70.91 (< 0.001)*
Plastron length	2.685 (< 0.001)*	70.587 (< 0.001)*
Plastron width	0.92 (0.45)	21.97 (< 0.001)*

**Fig. 3:**
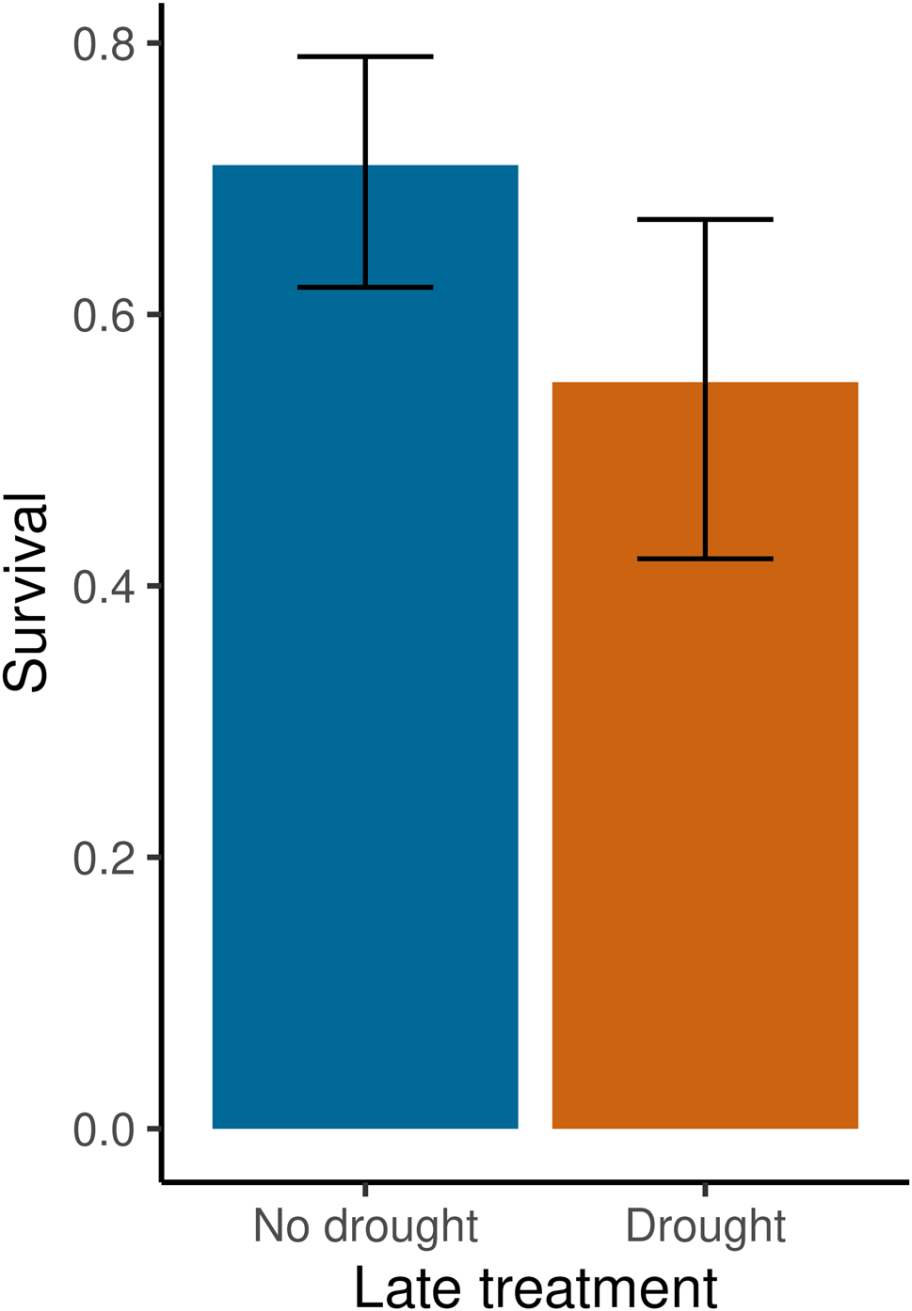
Predicted probability of recapture as a function of late incubation treatment with error bars representing 95% confidence intervals using a logistic regression model (N = 187, Z = -2.01, P = 0.045)

We recaptured 121 hatchlings out of 187 released (64.7%). Hatchling survival was adversely affected by drought exposure during the last three weeks of incubation (Z = −2.01, P = 0.045; Fig. 3); recapture rate was reduced by 22.5% compared to hatchlings that did not experience late drought. However, drought in the first 6 weeks of incubation had no effect on survival. Hatchling mass had no substantive effect on survival (Z = −0.60, P = 0.55), though there was a non-significant negative trend. Hatchling survival was similarly unaffected by carapace and plastron length and width (Table S4).

Hatchlings took 7–97 h to reach the drift fence (straight-line distance = 30 m). Average dispersal time was 49 ± 24 h. Drought treatment altered dispersal time (F = 4.3, P = 0.003). Hatchlings that experienced drought during mid-development took the longest to reach the fence, averaging 63 ± 26 h. Hatchling mass was unrelated to dispersal time (F = 0.022, P = 0.88). Recaptured hatchlings lost an average of 9.7% (1.06 ± 0.47 g) of their total body mass during dispersal. Drought treatment significantly affected mass lost (F = 3.06, P = 0.019), with hatchlings that experienced mid + late drought losing more mass than the control group relative to body size.

## Discussion

Flash droughts are increasing in frequency and intensity under climate change (Yuan et al. 2023), and their ecological impacts, particularly on vertebrates, are not well understood. In this study, we investigated the effects of flash drought at different stages of embryonic development in *Chelydra serpentina*, a ground-nesting oviparous vertebrate. We found that flash drought during the last two-thirds of embryonic development reduced egg mass, time to hatch, and four of five body size metrics. During hatchling dispersal from the nest, mid-incubation drought increased dispersal time, mid + late drought increased body mass loss, and late drought reduced survival. These results, which we explore in more detail below, indicate that flash drought, particularly during the final trimester of development, produces substantial and ecologically relevant effects on early-life offspring phenotypes and survival in this species.

Our finding that eggs decreased in size under drought conditions aligns with previous literature for this species (e.g., Janzen et al. 1990) and more broadly for other turtles and squamates with flexible-shelled eggs (Vleck 1991; Packard 1999; Delmas et al. 2008). We also found that drought during early incubation did not affect egg mass in the long term. This result supports the findings of some previous studies (Gutzke and Packard 1986; Delmas et al. 2008; but see Packard and Packard 1988). Eggs in the early drought group experienced an initial reduction in water potential, but when placed in a wet environment, they absorbed substrate water more rapidly than their counterparts in the control group, likely due to the larger difference in water potential relative to the substrate. This compensatory water exchange allowed them to replace mass lost during the first trimester over the ensuing six weeks (sensu Gutzke and Packard 1986).

Eggs that experienced drought during late and mid + late embryonic development had a slightly shorter incubation period. This result aligns with previous studies in turtles (Gutzke and Packard 1986; Packard and Packard 1988), but the mechanism of this phenomenon is not fully understood. Packard (1999) suggested that water-limited embryos are constrained in their growth, leading them to hatch earlier, while embryos with sufficient water reserves will continue to incubate and grow for a longer period of time. Regardless, the biological impact might be minimal given that treatment differences amounted to about 2 d at most (roughly 3% of overall incubation length).

In general, soil moisture is positively correlated with hatchling size in reptiles with flexible-shelled eggs, because greater water availability increases embryo metabolism and growth (Morris et al. 1983; Packard 1999). We found that this relationship is mediated by timing of hydric stress in *C. serpentina*. Hatchlings from eggs that experienced drought during mid-to-late embryonic development were smaller than those that experienced early or no drought. We attribute this result to the stages of embryonic development. The second and third trimesters of vertebrate embryogeny coincide with the organogenesis and growth phases of development, respectively (Yntema 1968). The differentiation of tissue and organ systems, and the subsequent considerable increase in body size, require substantial water intake for protein and calcium anabolism that may not be available in a drought environment (e.g., Janzen et al. 1990). This fundamental biochemistry and physiology may explain why drought during mid-to-late embryonic development produced smaller hatchlings.

In this study, flash drought during late embryonic development reduced hatchling survival during migration from the nest, whereas flash drought during early and mid-development did not. Our findings differ from two previous field studies that did not detect a relationship between constant embryonic water potential and post-hatching offspring fitness (Janzen 1993; Filoramo and Janzen 2002). However, hatchlings in the first study had access to water before the experimental release, while the “dry” treatment in the other study received more water than “wet” treatments in previous studies in the same species (Tucker and Paukstis 1999), suggesting that hatchlings may not have been experiencing significant water stress. Past studies have concluded that larger neonatal turtles have a survival advantage during dispersal (Janzen 1993; Janzen et al. 2000a, b; Janzen et al. 2007; Paitz et al. 2007; Tucker et al. 2008; but see Congdon et al. 1999; Kolbe and Janzen 2001; Filoramo and Janzen 2002; Colbert et al. 2022). However, our results do not appear to align with this pattern, as hatchling size had no detectable effect on survival during the release experiment. This outcome suggests that drought might affect offspring survival through another mechanism not considered in this study. One possibility could be differential body water content. Hatchlings migrating from the nest to water rely on the water reserve contained in their body tissues and yolk sac; those with lower body water content are more vulnerable to the negative physiological effects of dehydration (Finkler 1999). Another possibility could be changes to nitrogenous waste concentrations in the egg during embryonic development. Concentrations of urea are higher in *C. serpentina* eggs incubated in drier conditions (Packard et al. 1984), and hatchlings that experience hydric stress during incubation have higher levels of urea in blood plasma. Even at low levels, urea may inhibit metabolism, though it does not appear to affect hatchling size in this species (Packard and Packard 1989). Further research is needed to uncover the potential physiological mechanisms linking hydric stress and hatchling survival.

Future research could investigate the interactive effects of drought and other abiotic stressors, such as heat waves, on phenotype and fitness. Research is also needed to determine whether other turtle species, including those with rigid-shelled eggs, respond similarly to the timing of hydric stress compared to *C. serpentina*. Furthermore, studies of natural nests should be conducted in the field, to determine whether the results of our study are relevant in the more variable environmental conditions that occur in the field (Packard and Packard 2000). Finally, the physiological mechanisms underpinning the observed phenotypic and survival effects of flash drought during embryonic development should be explored. Future studies could examine, for example, which genes are being expressed differently under hydric stress, with a potential focus on those involved in muscle and bone growth.

Flash droughts are increasing in prevalence and intensity worldwide (Christian et al. 2021; Yuan et al. 2023), leading to higher levels of hydric stress for developing embryos. Our findings reveal that flash drought, particularly during mid-to-late embryonic development, can produce significant deleterious effects, including reduced body size and increased juvenile mortality in an oviparous vertebrate. This study highlights the importance of considering the developmental effects of acute hydric stress in the context of climate change.

## Supplementary Information

Below is the link to the electronic supplementary material.

Supplementary file1 (PDF 73 KB)

## Data Availability

Data will be available on the Dryad Digital Repository (10.5061/dryad.6hdr7sr9c).

## Code availability

R code used for the analysis will be available on Github (https://github.com/ayley-shortridge/Chelydra-serpentina).
