# Supplementary material for: Developmental timing of flash drought influences offspring survival in the field
Source: Oecologia. 2025 Apr 17;207(5):65. doi: 10.1007/s00442-025-05702-7 (PMC12006206; doi:10.1007/s00442-025-05702-7)
Supplement: Supplementary file 1 — Supplementary file1 (PDF 73 KB) [file 442_2025_5702_MOESM1_ESM.pdf]

Developmental timing of flash drought influences offspring survival in the field

Electronic supplementary material

Ayley L. Shortridge[1], Morgan A. Clark[1], Paulette Gutierrez[1], Caleb J. Krueger[2], Fredric J. Janzen[1,2]

[1]Ecology, Evolution, & Behavior Program, Department of Integrative Biology, W. K. Kellogg Biological Station, Michigan State University, Hickory Corners, MI, USA

[2]Department of Fisheries & Wildlife, W. K. Kellogg Biological Station, Michigan State University, Hickory Corners, MI, USA

Correspondence

Ayley L. Shortridge

Email: ayley.shortridge@gmail.com

Phone: (517) 316-5847

Table S1 Summary of egg and incubation data by clutch, with mean values ± 1 SD

Clutch	Date laid	Initial egg mass (g)	Hatching success	Time to hatch (d)
1	2 June	14.95 ± 0.60	0.95	60.4 ± 2.2
2	2 June	16.54 ± 0.61	0.95	60.0 ± 1.4
3	5 June	13.98 ± 0.58	1.00	61.4 ± 1.8
4	5 June	13.97 ± 0.77	0.90	62.4 ± 2.7
5	5 June	12.36 ± 0.45	0.95	60.8 ± 2.5
6	5 June	14.44 ± 0.57	0.90	61.0 ± 1.8
7	5 June	14.62 ± 0.46	0.95	61.0 ± 2.0
8	6 June	14.33 ± 0.48	1.00	61.3 ± 2.3
9	6 June	16.26 ± 0.32	0.95	62.3 ± 1.5
10	6 June	13.99 ±0.87	0.80	62.3 ± 1.6

Table S2 Summary of hatchling morphometrics by clutch, with mean values ± 1 SD

Clutch	Hatchling mass (g)	Carapace length (mm)	Carapace width (mm)	Plastron length (mm)	Plastron width (mm)
1	10.93 ± 0.85	31.57 ± 1.38	29.21 ± 1.34	21.66 ± 0.77	14.43 ± 0.78
2	12.47 ± 0.44	32.84 ± 1.11	31.04 ± 1.06	22.53 ± 0.85	15.21 ± 0.68
3	10.66 ± 0.69	32.42 ± 1.05	30.05 ± 1.23	21.48 ± 0.78	13.92 ± 0.72
4	10.66 ± 1.09	31.57 ± 1.67	30.01 ± 1.34	21.46 ± 1.21	13.63 ± 0.90
5	9.65 ± 0.76	30.87 ± 0.81	28.71 ± 1.28	20.59 ± 0.84	13.81 ± 0.76
6	11.32 ± 0.83	32.47 ± 0.87	30.39 ± 1.20	21.95 ± 0.87	14.12 ± 0.83
7	10.96 ± 0.89	32.88 ± 0.85	30.36 ± 1.27	21.47 ± 0.95	14.04 ± 0.83
8	11.03 ± 0.66	32.01 ± 0.89	28.83 ± 1.28	22.07 ± 0.88	14.59 ± 1.44
9	12.33 ± 0.95	33.42 ± 1.02	30.73 ± 1.03	22.41 ± 0.80	14.10 ± 0.97
10	10.30 ± 0.81	30.94 ± 1.57	28.90 ± 1.32	20.81 ± 0.81	13.63 ± 0.78

Table S3 Summary of release data by clutch, with mean values ± 1 SD

Clutch	Survival	Dispersal time (h)	Horizontal dispersal (m)	Mass lost (g)
1	0.53	58.0 ± 21.6	14.0 ± 8.8	1.07 ± 0.39
2	0.58	45.2 ± 22.0	10.9 ± 7.4	1.51 ± 0.51
3	0.65	55.5 ± 26.8	11.9 ± 9.5	0.99 ± 0.48
4	0.78	41.3 ± 25.3	15.7 ± 10.2	0.95 ± 0.31
5	0.63	43.0 ± 26.2	15.4 ± 15.1	0.78 ± 0.50
6	0.72	50.9 ± 29.4	21.5 ± 13.3	1.09 ± 0.53
7	0.68	55.5 ± 28.4	12.7 ± 9.3	1.11 ± 0.41
8	0.55	44.1 ± 20.9	15.0 ± 10.7	1.06 ± 0.42
9	0.74	49.4 ± 26.8	12.5 ± 9.1	0.96 ± 0.37
10	0.62	52.6 ± 27.2	16.0 ± 8.8	1.18 ± 0.60

Table S4 Logistic regression of the effects of drought, hatchling mass, and four metrics of body size on recapture

Source of variation	Estimate	Standard Error	Z-value	P-value
Early treatment	0.168	0.402	0.416	0.68
Middle treatment	0.316	0.329	0.963	0.34
Late treatment	-0.712	0.355	-2.009	0.045*
Hatchling mass	-0.081	0.136	-0.595	0.55
Carapace length	0.015	0.111	0.137	0.89
Carapace width	0.010	0.106	0.092	0.93
Plastron length	-0.060	0.147	-0.411	0.68
Plastron width	-0.130	0.155	-0.842	0.40
